# Extracellular DNA Chelates Cations and Induces Antibiotic Resistance in *Pseudomonas aeruginosa* Biofilms

**DOI:** 10.1371/journal.ppat.1000213

**Published:** 2008-11-21

**Authors:** Heidi Mulcahy, Laetitia Charron-Mazenod, Shawn Lewenza

**Affiliations:** Department of Microbiology and Infectious Diseases, University of Calgary, Calgary, Alberta, Canada; Schepens Eye Research Institute, United States of America

## Abstract

Biofilms are surface-adhered bacterial communities encased in an extracellular matrix composed of DNA, bacterial polysaccharides and proteins, which are up to 1000-fold more antibiotic resistant than planktonic cultures. To date, extracellular DNA has been shown to function as a structural support to maintain *Pseudomonas aeruginosa* biofilm architecture. Here we show that DNA is a multifaceted component of *P. aeruginosa* biofilms. At physiologically relevant concentrations, extracellular DNA has antimicrobial activity, causing cell lysis by chelating cations that stabilize lipopolysaccharide (LPS) and the outer membrane (OM). DNA-mediated killing occurred within minutes, as a result of perturbation of both the outer and inner membrane (IM) and the release of cytoplasmic contents, including genomic DNA. Sub-inhibitory concentrations of DNA created a cation-limited environment that resulted in induction of the PhoPQ- and PmrAB-regulated cationic antimicrobial peptide resistance operon *PA3552–PA3559* in *P. aeruginosa*. Furthermore, DNA-induced expression of this operon resulted in up to 2560-fold increased resistance to cationic antimicrobial peptides and 640-fold increased resistance to aminoglycosides, but had no effect on β-lactam and fluoroquinolone resistance. Thus, the presence of extracellular DNA in the biofilm matrix contributes to cation gradients, genomic DNA release and inducible antibiotic resistance. DNA-rich environments, including biofilms and other infection sites like the CF lung, are likely the *in vivo* environments where extracellular pathogens such as *P. aeruginosa* encounter cation limitation.

## Author Summary

*Pseudomonas aeruginosa* is an opportunistic pathogen, which causes a variety of serious infections in immunocompromised patients and cystic fibrosis (CF) sufferers. The biofilm-forming ability of *P. aeruginosa* is thought to contribute to chronic *P. aeruginosa* infection of the CF lung. Biofilms are dense communities of bacteria, encased in an extracellular matrix, that are practically impossible to eradicate using available antimicrobial therapies. Understanding the mechanisms by which biofilm bacteria develop resistance to antibiotics is paramount to expanding the treatment options available to patients with chronic biofilm infections. In this study we have identified a novel mechanism of biofilm-specific antibiotic resistance. Extracellular DNA, a known component of biofilms, was found to induce antibiotic resistance. This previously unidentified function of DNA was due to its ability to bind and sequester cations, including magnesium, from the surrounding environment. This environmental cue was then detected by *P. aeruginosa* leading to induction of genes involved in modification of the cell surface component, lipopolysaccharide (LPS), resulting in physical alterations in the bacterial outer membrane (OM). These results demonstrate a novel function for DNA in biofilms and identify cation chelation by DNA as a previously unrecognized mechanism, which can explain the increased resistance of biofilms to antimicrobial agents.

## Introduction

*Pseudomonas aeruginosa* is an opportunistic pathogen capable of causing both acute and chronic infections. It is the third-leading cause of nosocomial infections and is the predominant pathogen associated with morbidity and mortality of CF patients [1],[2]. The biofilm-forming ability of *P. aeruginosa*, and indeed other bacteria, is thought to contribute to their ability to thrive in hostile host environments and result in chronic infection [3],[4].

Biofilms are multicellular surface-associated microbial communities encased in an extracellular matrix which display a characteristic structure and increased resistance to antimicrobial compounds and environmental stresses. *P. aeruginosa* biofilms are up to 1000-fold more antibiotic tolerant than planktonic cells, to single and combination antibiotics [5]–[7]. As acute CF exacerbations caused by *P. aeruginosa* are often treated with combination antibiotic therapy [8]–[10], the increased resistance of biofilms to combination antibiotics is of direct clinical relevance.

Eighty five percent of *P. aeruginosa* strains isolated from the lungs of CF patients with advanced stages of disease have a distinctive mucoid colony morphology [11]. This mucoid phenotype is a result of overproduction of the alginate exopolysaccharide (EPS) [1],[12]. Alginate production has been shown to inhibit phagocytic killing of *Pseudomonas*, to protect from antibiotic exposure [13],[14], and is associated with poor prognosis for the infected patients [15],[16]. The direct observation of *P. aeruginosa* microcolonies encased in an alginate matrix in microscopy studies of CF bronchial samples [17], along with a large body of additional *in vitro* and *in vivo* data [7], [18]–[21] suggests that *P. aeruginosa* forms biofilms in the lungs of CF patients.

The mechanisms of biofilm-associated antibiotic resistance are distinct from the well studied intrinsic resistance mechanisms such as drug efflux, drug inactivation, membrane permeability and target site alterations. Although the basis of biofilm-associated antibiotic resistance is not fully understood, it is likely that multiple mechanisms operate simultaneously in biofilms to contribute to antibiotic resistance. Cells in a biofilm may be protected from antibiotic exposure due to the restricted penetration of antibiotics through the biofilm matrix [19]. However, while the biofilm matrix may limit diffusion initially for certain antibiotics such as β-lactams and aminoglycosides [14],[22], the penetration of fluoroquinolones occurs immediately and without delay [23]–[25]. The rate of diffusion through the matrix is presumably dependent on binding of the antibiotic molecules to the EPS matrix. Once the matrix becomes saturated, diffusion and antimicrobial activity of the drug will resume [26]. It is the general consensus that reduced diffusion through the biofilm matrix only provides a short-term protective effect and does not play a significant role during long-term antibiotic exposure [26].

Other resistance mechanisms include the presence of subpopulations of multidrug tolerant persister cells [27]–[29], drug indifference of slow-growing, nutrient-limited cells [30], and unique resistance mechanisms specifically associated with biofilms [31],[32]. Despite the fact that biofilms are recognized as the predominant mode of bacterial growth in nature and are responsible for the majority of refractory bacterial infections [19], little is known regarding the mechanisms of biofilm-specific antibiotic resistance. Furthering our understanding of the mechanisms underlying biofilm-associated antibiotic resistance will significantly improve the treatment options available to patients with chronic bacterial infections.

Signal transduction systems have been documented to be involved in the regulation of biofilm formation in multiple bacterial species including *P. aeruginosa*, *S. aureus*, *E. coli* and *V. fischeri* [33]–[38]. These two component systems (TCS) are comprised of an membrane-anchored histidine kinase sensor and a cytoplasmic response regulator. After detecting specific environmental signals, a signal transduction cascade is initiated that results in phosphorylation of the response regulator, which activates or represses the necessary target genes. A number of regulatory systems that influence biofilm formation have been described. These include, but are not limited to, the global virulence factor regulator GacA, mutation of which results in a 10-fold decrease in biofilm formation and failure to form microcolony structures [33]. Additionally, the hybrid sensor kinases, LadS and RetS appear to work upstream of GacA to possibly control the switch to a biofilm lifestyle [34],[35]. Mutations in *algR*, a response regulator protein required for synthesis of alginate, which is a major component of the matrix of biofilms in the cystic fibrosis lung [1] results in a *P. aeruginosa* strain that has decreased type IV pili-dependent motility and biofilm formation [39]. The three-component system SadARS which regulates the formation of mature microcolonies [40] and PvrR, a response regulator involved in the switch from planktonic to antibiotic-resistant biofilm cells in *P. aeruginosa* are additional examples of regulators of biofilm formation [41].

During the course of an infection, one of the first lines of defense encountered by colonizing bacteria is the production of cationic antimicrobial peptides (CAPs) by a variety of host cells including neutrophils, platelets and epithelia. CAPs are short, amphipathic peptides that bind to and disrupt both the outer and cytoplasmic membranes resulting in cell death. The broad-spectrum antimicrobial activity of CAPs against Gram-negative and Gram-positive bacteria accounts for their role as an essential component of the innate immune response of humans, animals and insects. Cationic peptides, which have antimicrobial and immunomodulatory activities, are being developed as a promising new class of therapeutically relevant drugs [42].

In *P. aeruginosa*, resistance to CAPs is inducible by the PhoPQ and PmrAB TCSs, both of which are activated independently in response to limiting Mg^2+^ [43]–[46]. Under conditions of limiting magnesium, PhoP and PmrA bind to the promoter of the CAP resistance operon *PA3552–PA3559* (*arnBCADTEF-ugd*) and induce its expression [45]–[47]. These genes encode an LPS modification pathway required for the addition of aminoarabinose to lipid A, which reduces the OM permeability to CAPs [48]. The PhoPQ and PmrAB regulatory systems are well studied in planktonic cultures and have been shown to induce modest resistance to CAPs (8-fold) under low Mg^2+^ conditions [45]. However, while the *PA3552–PA3559* operon has been reported to be expressed in biofilms cultivated in flowcells, and is required for survival in response to colistin treatment [49], little else is known regarding these systems and the role they may play in biofilm-associated antibiotic resistance.

The extracellular matrix of *P. aeruginosa* biofilms includes extracellular DNA [50],[51], multiple bacterial exopolysaccharides and host proteins [4],[52]. Extracellular DNA, which is a matrix component of both Gram-positive and Gram-negative bacterial biofilms [51],[53], functions to maintain the 3D biofilm architecture by acting as a cell-cell interconnecting compound [50]. Genomic DNA has been shown to localize to the biofilm surface, surrounding the mushroom-shaped microcolonies [51]. DNA in the biofilm matrix is likely released by dead bacteria or immune cells. It has been reported that prophage-mediated cell death is an important mechanism in the differentiation and dispersal of biofilms [54],[55]. Additional sources of DNA in biofilms may include the quorum sensing regulated release of DNA [51] and/or DNA contained within outer membrane vesicles (OMV) that bleb and are released from the OM of living *P. aeruginosa* cells [56],[57]. Furthermore, while a specific mechanism of DNA release has not been reported for *P. aeruginosa* it is possible that such a method may exist, similar to the autolysin-mediated DNA release observed in *Staphylococcus epidermidis* biofilms [53].

In this study we sought to examine if the presence of DNA in biofilms may contribute to biofilm-specific antibiotic resistance. Here we identify a novel cation chelating property of DNA, which has several important consequences for biofilm physiology and antibiotic resistance in biofilms.

## Results

### Extracellular DNA has antimicrobial activity

**Figure 1:**
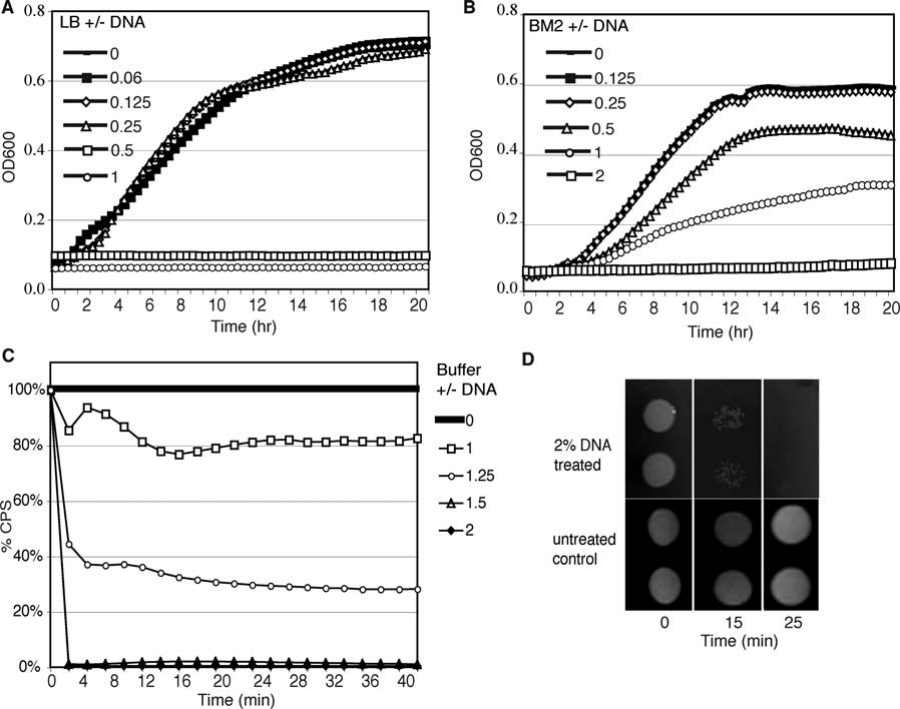
Extracellular DNA inhibits planktonic growth by rapidly killing *Pseudomonas aeruginosa*. Growth of PAO1 in (A) LB or (B) BM2 media in cultures supplemented with % (w/v) extracellular salmon sperm DNA, as indicated. Growth (OD600) was measured every 20 minutes, over 20 h. (C) Overnight cultures of PAO1::p16s*lux* were washed and 5×10^7^ cfu resuspended in sodium phosphate buffer (25 mM, pH 7.4). Resuspended cells were treated with varying concentrations of salmon sperm DNA, as indicated, and luminescence was measured in cps (counts per second) over time, as a measure of viability. Cells were also resuspended in buffer +0% (w/v) DNA as a negative control. Data is expressed as percentage survival relative to the untreated control. For experiments 1A–C the mean of three replicate experiments is represented. The standard deviation, omitted for clarity, was not greater than +/−10% the mean. (D) The loss of viability of PAO1::p16S*lux*, following 2% (w/v) DNA treatment, was confirmed by stamping of cultures at indicated time points post-treatment on LB agar. Cells resuspended in buffer in the absence of DNA remained viable. Two replicate wells are shown for each condition.

To study the role of the matrix component DNA on biofilm formation and biofilm-associated antibiotic resistance, we first examined the influence of extracellular DNA on *P. aeruginosa* growth in rich and defined media, LB and BM2, respectively. Addition of 0.5% (w/v) (5 mg/ml) or greater extracellular DNA to LB or 1% (w/v) or greater DNA to BM2 media inhibited growth of *P. aeruginosa* (Fig 1A and 1B). The kinetics of killing by extracellular DNA was determined by measuring the loss of luminescence from a chromosomally-tagged luminescent *P. aeruginosa* strain, PAO1::p16S*lux*. DNA-mediated killing was fast, within minutes, as measured by the rapid loss of luminescence upon exposure to 1.25% (w/v) DNA, or greater (Fig 1C). Killing was dose-dependent, with faster killing observed as the DNA concentration increased (Fig 1C). The rapid decrease in luminescence corresponded with a loss of bacterial viability, as determined by plating (Fig 1D).

One percent (w/v) extracellular DNA in LB also inhibited the growth of *Escherichia coli*, *Staphylococcus aureus* and *Burkholderia cenocepacia* (data not shown), suggesting that the antimicrobial activity of DNA is not unique to *P. aeruginosa*.

### Extracellular DNA induces cell death by membrane perturbation and cell lysis

DNA is a highly anionic polymer due to the phosphates in the deoxyribose backbone. This property, in combination with the fast-killing observed in response to extracellular DNA led us to hypothesize that addition of exogenous DNA resulted in the loss of membrane integrity through cation chelation, in a manner similar to that observed with the known cation chelator EDTA [58]. The OM of *P. aeruginosa* contains a 20∶1 ratio of Mg^2+^∶Ca^2+^ cations [59], which bind to and stabilize LPS in the outer leaflet of the OM [58]. EDTA treatment of cells resulted in chelation and removal of divalent cations from the OM, leading to disruption of the OM [58]. To determine the effect of DNA on membrane integrity, microscopic analysis in response to lethal concentrations of DNA and relevant controls was performed.

**Figure 2:**
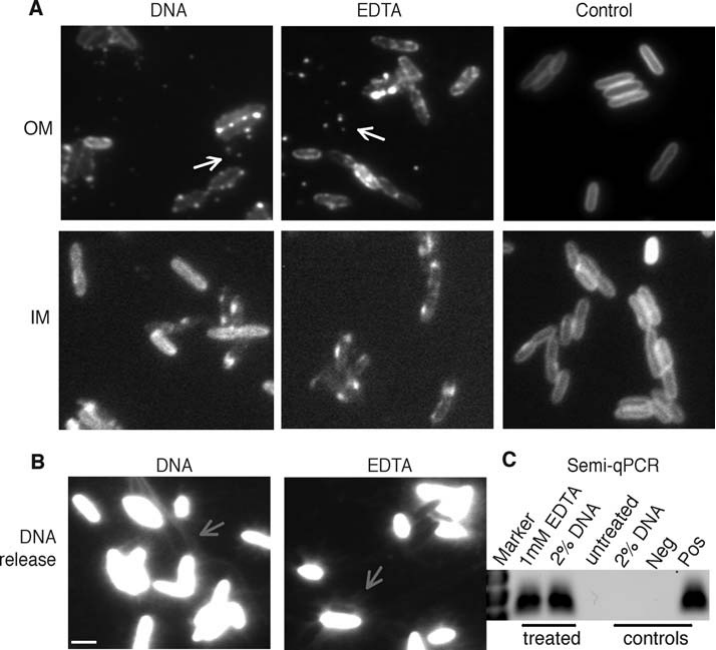
Extracellular DNA induces membrane perturbation, cell lysis, DNA release and death. (A) Membrane integrity was monitored by visualizing DNA, EDTA or buffer treated *P. aeruginosa* producing mCherry fluorescent lipoproteins in either the IM or OM. The release of OMVs (white arrow) and genomic DNA strands (grey arrow) into the extracellular milieu following cell lysis in response to DNA or EDTA treatment was demonstrated by (B) PI staining and (C) semi-quantitative PCR which detects *P. aeruginosa* genomic DNA but not salmon sperm DNA. Cells were treated with 2% (w/v) salmon sperm DNA, 2 mM EDTA, or buffer alone (negative control), pelleted and 1 µl of supernatent used as a template for semi-quantitative PCR. PCR controls included 2% (w/v) salmon sperm DNA (primer specificity) and a negative and positive PCR control. The scale bar equals 2.5 microns.

Lipoproteins are lipid-modified proteins anchored in the outer leaflet of the IM or the inner leaflet of the OM. *P. aeruginosa* cells producing mCherry fluorescent membrane-anchored lipoproteins (lipoChFP) that are localized to either the OM or IM [60],[61] were used as markers of OM and IM integrity. LipoChFP-labelled *P. aeruginosa* cells showed dramatic membrane perturbations when exposed to 2% (w/v) DNA, but showed uniform membrane staining patterns in untreated cells (Fig 2A). The OM perturbations in DNA-exposed cells included regions of patchy fluorescence and the release of OMVs, while the IM perturbations were visualized simply as patchy and irregular regions of membrane fluorescence (Fig 2A). EDTA, the known cation chelator caused comparable IM and OM perturbations as those observed in cells exposed to extracellular DNA. Propidium iodide (PI) stains extracellular DNA and DNA in dead cells. PI staining was observed in cells exposed to DNA and EDTA, confirming that this treatment was lethal (Fig 2B). PI staining also revealed the presence of long strands of genomic DNA, presumably as a consequence of the loss of membrane integrity, cell lysis and release of cytoplasmic contents, including DNA (Fig 2B). The DNA released by lysed cells formed a mesh-like coating surrounding and connecting individual bacterial cells (Fig 2B). Degradation of these strands by DNAse treatment of lysed cells confirmed that these fibres were composed of DNA (Fig S1). *Pseudomonas* specific semi-quantitative PCR (qPCR) was also performed to confirm that the DNA released following DNA or EDTA treated cells was in fact genomic DNA from *P. aeruginosa* (Fig 2C). Buffer treated control cells showed intense green staining with syto9 (indicating viability) and a lack of PI staining (indicating no dead/dying cells or DNA release) (Fig S1).

### DNA has cation chelating activity

**Figure 3:**
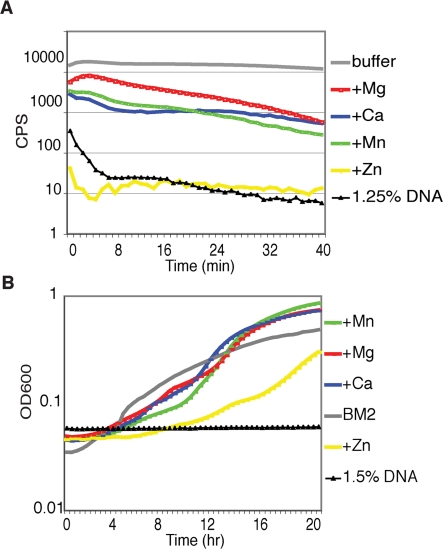
DNA induces cell death by acting as a cation chelator. (A) Killing assays indicate relative protection provided by cations following pre-incubation of salmon sperm DNA with cations (25 mM Mg^2+^; 6.25 mM Ca^2+^; 6.25 Mn^2+^; 3.125 mM Zn^2+^) in Hepes buffer (50 mM, pH 7.4). (B) Restoration of PAO1 growth in BM2 supplemented with 1.5% (w/v) DNA following addition of excess individual cations (10 mM Mg^2+^, 10 mM Ca^2+^, 0.5 mM Mn^2+^, 2.5 mM Zn^2+^). Each experiment was performed at least five times and representative curves are shown. Standard deviations for each experiment were not greater than +/−10% of the value shown.

The observation that DNA disrupted the integrity of the cell envelope causing cell lysis suggested that DNA was acting as a cation chelator. To confirm that DNA-mediated killing was a result of cation chelation, excess Mg^2+^, Ca^2+^, Mn^2+^, and Zn^2+^ were added to *P. aeruginosa* cultures. The rapidity of DNA-induced cell death ruled out the possibility that death, or lack of growth, was simply due to cation starvation. Addition of excess cations to planktonic cultures inhibited the fast-acting antimicrobial effects of DNA (Fig 3A). Protection was measured in response to a range of cation concentrations, where the highest concentration tested was that which remained soluble in the presence of DNA (3.125–25 mM). The concentration at which maximal protection was obtained for each cation is represented in Fig 3A (25 mM Mg^2+^; 6.25 mM Ca^2+^; 6.25 Mn^2+^; 3.125 mM Zn^2+^). Kill curve assays indicated that the addition of Mg^2+^, Ca^2+^ or Mn^2+^ provided protection from DNA-induced lysis, however, the addition of Zn^2+^ did not protect from DNA-mediated killing (Fig 3A). In a similar manner, the addition of excess Mg^2+^, Ca^2+^ and Mn^2+^ restored growth of *P. aeruginosa* in BM2 media. Only partial restoration of growth was observed in the presence of Zn^2+^ (Fig 3B). The increased protection observed following addition of Mg^2+^ and Ca^2+^ is likely due to their importance in membrane integrity where they function to stabilize the OM by crosslinking adjacent LPS molecules [58].

Cations play diverse physiologically important roles within a cell; from detoxification of reactive oxygen species and co-factors for enzymes to the stabilization of macromolecules within the cell [62]. Since Mg^2+^ limitation has been shown to be associated with CAP resistance in *P. aeruginosa* [44],[45],[47], we sought to determine if Mg^2+^ chelation by DNA may account, at least in part, for the increased antibiotic resistance observed in biofilms.

### Extracellular DNA induces expression of the *PA3552–PA3559* CAP resistance operon in planktonic cultures

**Figure 4:**
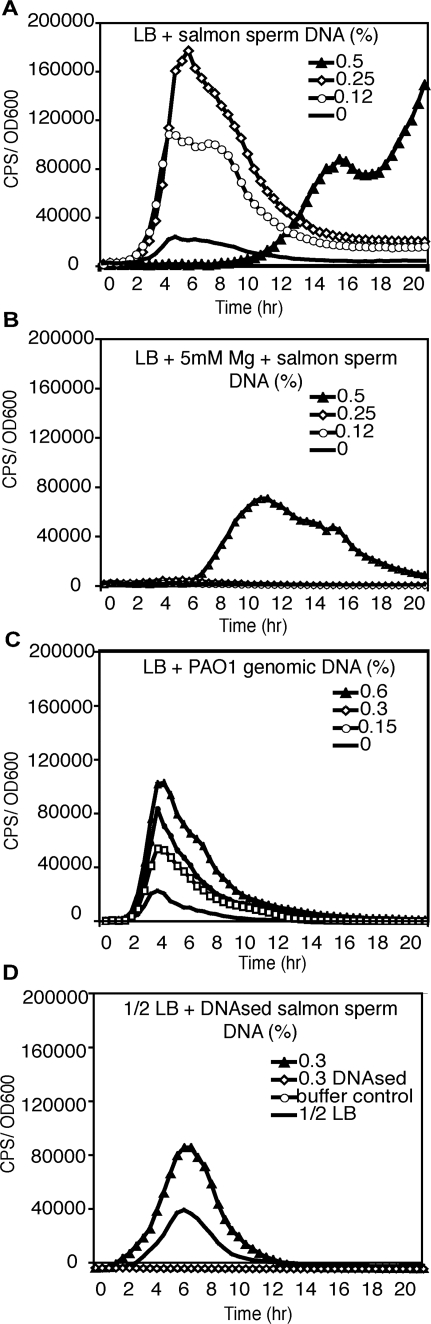
Extracellular DNA induces *PA3553* gene expression in planktonic cultures. Effects of (A) salmon sperm DNA, (B) salmon sperm DNA in the presence of excess (5 mM) Mg^2+^ (C) sonicated *P. aeruginosa* PAO1 genomic DNA and (D) DNAsed salmon sperm DNA on the expression of the *PA3553::lux* transcriptional fusion in planktonic cultures. Gene expression was normalized to growth for each condition and CPS/OD600 values are presented. Each growth experiment was performed at least five times and representative curves are shown. (D) Buffer control indicates that this sample was treated identically to the DNAsed DNA sample except for the addition of DNAseI enzyme. Standard deviations for each experiment were not greater than +/−10% of the value shown.

In *P. aeruginosa*, the PhoPQ and PmrAB-controlled response to magnesium limitation includes the induction of the PA3552 and its neighbouring genes. The genes *PA3552–PA3559* are co-regulated under low Mg^2+^ conditions and are thought to be organized as an operon [45]–[47]. These genes encode an LPS modification pathway required for the addition of aminoarabinose to lipid A, which reduces the OM permeability to CAPs, thus conferring resistance [48]. To determine if extracellular DNA imposes Mg^2+^ limitation, we measured the gene expression of a chromosomally encoded transcriptional *lux* (bioluminescence) fusion to *PA3553*, as a measure of the CAP resistance operon expression in planktonic cultures. *PA3553::lux* expression was strongly induced (up to 10-fold) by sub-inhibitory concentrations of low molecular weight salmon sperm DNA (Fig 4A). Induction of the CAP resistance operon was dose-dependent with increasing DNA concentrations up to 0.5% (w/v) DNA, at which growth is inhibited (Fig 4A). Addition of excess Mg^2+^ (5 mM) to the growth medium completely repressed the expression of *PA3553* in cultures supplemented with DNA, except at the highest DNA concentration tested (0.5% (w/v)) (Fig 4B). A similar induction profile of *PA3553::lux* was observed following exposure to high molecular weight *P. aeruginosa* genomic DNA (not shown) or *P. aeruginosa* genomic DNA that was mechanically sheared by sonication (Fig 4C). *P. aeruginosa* genomic DNA inhibited growth at similar concentrations as salmon sperm DNA. Thus, the ability of extracellular DNA to chelate magnesium is independent of origin and molecular weight, indicating that chelation is a general property of this negatively charged polymer. To ensure that induction of *PA3553* expression was specific to the ability of DNA to chelate cations, DNAse treated DNA was assessed for its ability to induce *PA3553* gene expression (Fig 4D). DNAse treated DNA failed to induce *PA3553* gene expression. However the addition of DNAse buffer to cells in our buffer control experiment also abolished induction of *PA3553*. This is due to the addition of excess Mg^2+^ ions as part of the DNAse buffer, which is required for DNAse activity. Thus, it is impossible to determine conclusively if DNAse treatment of DNA abolished *PA3553* gene expression.

### Expression of the CAP resistance operon *PA3552–PA3559* is induced in biofilms in response to extracellular DNA

**Figure 5:**
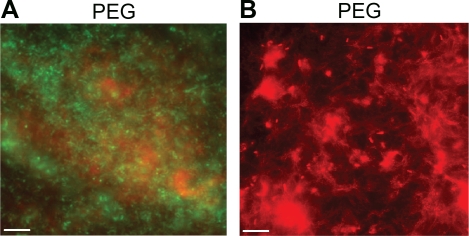
Visualization of DNA as a component of peg-adhered biofilms. (A) The extracellular stain DDAO demonstrated DNA is a component of PAO1 biofilms cultivated in BM2 on pegs (40× magnification). (B) PI staining indicates the presence of DNA as a mesh-like pattern in 1 day-old biofilms (right panel) (40× magnification). The scale bar equals 10 microns. Images presented are representative of triplicate experiments.

To determine the influence of extracellular DNA on *PA3553* gene expression in biofilms, DNA-enriched biofilms were cultivated on the surface of polystyrene pegs. Consistent with previous reports that DNA is a component of biofilms [50],[51], we observed DNA in 24 h old peg-adhered biofilms (Fig 5A and 5B). Double staining of *P. aeruginosa* with syto9 (stains viable cells green) and the extracellular DNA stain DDAO (red) [51] was used to visualize DNA as a loose lattice in biofilms formed on polystyrene pegs after 24 h (Fig 5A). DNA was also visualized (PI stained) as a mesh-like DNA matrix in 1 day-old peg-adhered biofilm monolayers (Fig 5B), which resembled the thread-like projections of genomic DNA observed in DNA or EDTA-lysed cells (Fig 2B). These localization patterns of extracellular DNA are suggestive of DNA gradients within biofilms.

**Figure 6:**
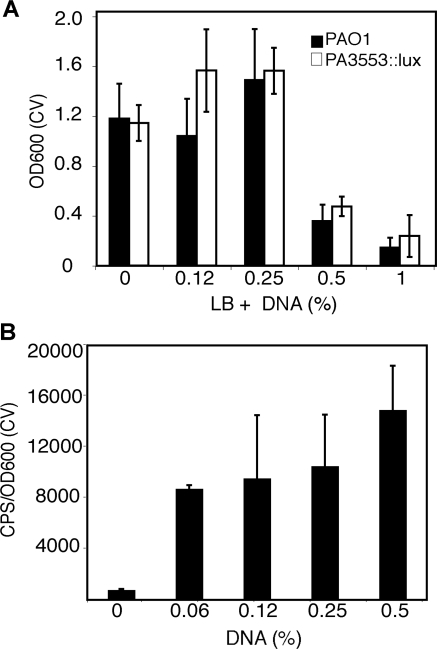
Extracellular DNA induces *PA3553* gene expression in peg-adhered biofilms. (A) Adhesion of PAO1 and *PA3553::lux* to polystyrene pegs assessed by crystal violet staining and OD600 measurement at 24 h in cultures supplemented with salmon sperm DNA, as indicated. (B) Gene expression from *PA3553::lux* was monitored in peg-adhered biofilms at 24 h in DNA supplemented conditions, as indicated. CPS values were normalized to the number of peg-adherent cells (OD600 of CV staining). Bars in (A) and (B) represent the average values obtained from eight pegs and the error bars represent the standard deviations.

Biofilm formation was inhibited at extracellular DNA concentrations ≥0.5% (w/v) (Fig 6A). This is consistent with the observed growth inhibition of planktonic cells at similar DNA concentrations (Fig 1A). One-day old *PA3553::lux* biofilms were washed to remove non-adhered cells and gene expression was measured from the cells adhered to the polystyrene peg surface. *PA3553* gene expression was strongly induced, up to 20-fold, in peg-adhered biofilms, with the highest induction at 0.5% (w/v) extracellular DNA (Fig 6B). Although gene expression was measured in a mutant background, both PAO1 and *PA3553::lux* had similar biofilm phenotypes in each condition tested (Fig 6A). In biofilms cultivated in the presence of extracellular DNA supplemented with excess Mg^2+^ (5 mM), *PA3553* gene expression was completely repressed (data not shown).

### DNA chelation of Mg^2+^, Ca^2+^ or Mn^2+^ but not Zn^2+^ induces *PA3553* expression

**Figure 7:**
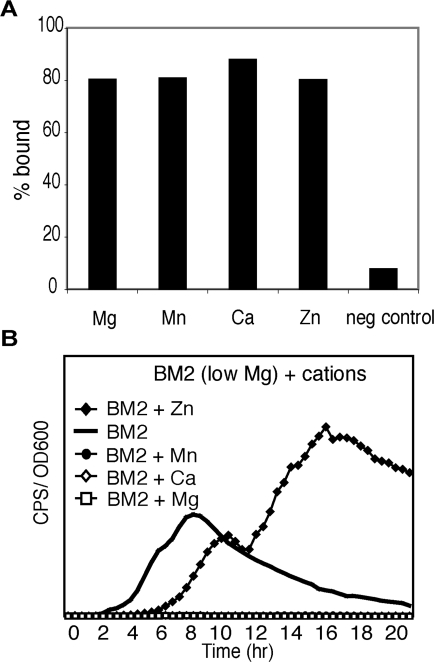
Multiple cations are bound by DNA and repress the induction of *PA3553* gene expression. (A) Elemental analysis of cations in buffer after size-exclusion centrifugation to remove DNA illustrates percentage bound to DNA. Values presented represent the % (w/v) bound to DNA relative to the total amount of cation added. The negative control indicated relates to the concentration of Mg^2+^ that bound to the column in the absence of DNA. (B) The influence of excess Mg^2+^, Ca^2+^, Mn^2+^ or Zn^2+^ on *PA3553* gene expression when grown in BM2 media with low (20 µM) Mg^2+^. Cations are added at concentrations of 5 mM, 5 mM, 1 mM and 2.5 mM, respectively. A representative curve from triplicate experiments is shown. Standard deviations for each experiment were not greater than +/−10% of the value shown.

At lethal concentrations, extracellular DNA induced cell lysis by chelating cations from the OM. This antimicrobial activity can be prevented if DNA is pre-loaded with Mg^2+^, Ca^2+^ or Mn^2+^, but not Zn^2+^, prior to treatment of cells (Fig 3A and 3B). To determine the specificity of cation chelation, flame atomic absorption spectroscopy was employed to quantitate DNA-dependent removal of cations from buffer containing known concentrations of Mg^2+^, Ca^2+^, Mn^2+^ or Zn^2+^ and a combination of all four cations. DNA was capable of binding all four cations at similar levels (80–88%), whether alone (Fig 7A) or in combination (data not shown). To ensure binding was specific to DNA a negative control was included. The concentration of Mg^2+^ that bound to the column in the absence of DNA is indicated.

At sub-lethal concentrations, extracellular DNA imposes a cation limitation that leads to induction of *PA3553* (Fig 4A), which can be repressed by excess Mg^2+^ (Fig 4B), indicating that *P. aeruginosa* senses Mg^2+^. The *P. aeruginosa* PhoQ sensor kinase protein has been shown to bind to and be repressed by Mg^2+^ and Ca^2+^ cations [63],[64]. Under limiting Mg^2+^ conditions, the addition of excess Mg^2+^, Ca^2+^ or Mn^2+^, but not Zn^2+^, repressed *PA3553* expression (Fig 7B). Taken together, these data indicate that *P. aeruginosa* can sense the presence of Mg^2+^, Ca^2+^ or Mn^2+^ and that chelation of these same cations by DNA results in induction of the *PA3552–PA3559* LPS modification operon.

### DNA induces resistance to CAPs and aminoglycosides

To determine if DNA-induced expression of *PA3552–PA3559* resulted in increased resistance to antimicrobials, antibiotic susceptibility testing of *P. aeruginosa* biofilms grown in the presence and absence of extracellular DNA was performed. Biofilms were cultivated on 96-well format, polystyrene pegs submerged in BM2 defined media, with or without sub-inhibitory concentrations of extracellular DNA (0.75% (w/v)), and challenged with antibiotics. After antibiotic challenge, this assay allows for determination of both the minimum inhibitory concentration (MIC) of planktonic cultures and the minimum biofilm eradication concentration (MBEC).

Consistent with previous results reporting on the antibiotic resistance phenotype of bacterial biofilms [6],[19], the MBEC values of biofilms cultivated in magnesium-replete conditions and treated with CAPs (polymyxin B, colistin) or aminoglycosides (gentamycin, tobramycin) were up to 64-fold higher than the MIC values of planktonic cultures (Table 1). The MBEC values of biofilms supplemented with extracellular DNA were 8 and 64-fold more CAP and aminoglycoside resistant than biofilms without exogenous DNA, respectively (Table 1). DNA-enriched biofilms were dramatically more resistant than planktonic cultures, up to 256-fold, and this resistance phenotype to CAPs and aminoglycosides was also observed in planktonic cultures supplemented with DNA. The simple addition of sub-inhibitory DNA amounts to planktonic cultures closely simulated the resistance-inducing effects of DNA in a biofilm (Table 1).

**Table 1:** Influence of extracellular DNA on antibiotic resistance in biofilms.

Strain	Cultivation and challenge conditions^a^	MIC^b^				MBEC^c^			
		PxnB	Coln	Gm	Tm	PxnB	Coln	Gm	Tm
PAO1	−DNA	10	10	5	1.25	640	320	20	5
PAO1	+DNA	1280	320	1280	320	2560	2560	1280	640
*PA3553::lux*	−DNA	10	10	5	1.25	640	640	10	10
*PA3553::lux*	+DNA	20	20	10	5	320	320	320	160

a Biofilms were cultivated and challenged in BM2 defined medium (2 mM Mg^2+^) with or without 0.75% (w/v) salmon sperm DNA, as indicated.

b The minimum inhibitory concentration (MIC) is defined as the lowest concentration of antibiotic required to inhibit the growth of planktonic cells that were shed from the peg.

c The minimum biofilm eradication concentration (MBEC) is defined as the lowest concentration of antibiotic required for complete killing of the surface-adhered biofilm. The MIC and MBEC values shown are the median values from four replicate experiments.

PxnB = polymyxin B; Coln = colistin; Gm = gentamycin; Tm = tobramycin.

The MIC values for polymyxin B and gentamicin are equal to 1 µg/ml and 2 µg/ml, respectively, using the standard microbroth dilution method for antimicrobial susceptibility testing (National Committee on Clinical Laboratory Standards (NCCLS) protocol) (data not shown). Thus, depending on the method used to determine the MIC (CBD or NCCLS protocol), DNA-enriched biofilms can be up to 2560-fold more polymyxin B resistant and up to 640-fold more aminoglycoside resistant than planktonic cultures. DNA-enriched biofilms did not show an increased tolerance to ceftazidime (β-lactam) or ciprofloxacin (fluoroquinolone) (data not shown). Since extracellular DNA is a natural matrix component of PAO1 biofilms (Fig 5A and 5B), DNA-induced antibiotic resistance is likely to be a phenomenon unique to biofilms or other DNA rich environments. The presence of DNA in peg-cultivated biofilms (Fig 5A), grown in the absence of exogenous DNA, likely contributes to the increased antibiotic resistance generally observed in biofilms (Table 1).

We have shown previously that the *PA3552–PA3559* operon is required for resistance to cationic antimicrobial peptides in planktonic cultures grown in limiting magnesium conditions [47]. To determine if DNA-induced resistance requires these genes in biofilms, the resistance phenotype of the *PA3553::lux* mutant was determined. *PA3553::lux* had no significant DNA-induced CAP resistance in biofilm or planktonic cultures, confirming that these genes are essential for CAP resistance in the presence of extracellular DNA (Table 1). The *PA3553* mutant also displayed decreased DNA-induced resistance to aminoglycosides compared to PAO1. The differences observed between CAP and aminoglycoside resistance in *PA3553::lux* suggests that DNA-induced resistance to aminoglycosides is not limited to *PA3553* induction. The biofilms formed by the *PA3553::lux* mutant were unaltered compared to PAO1 biofilms under these conditions, ensuring that the difference observed was not due to an altered biofilm phenotype (Fig 6B).

The CAP resistance phenotype of biofilms grown in limiting magnesium (20 µM) was similar to biofilms grown in DNA, confirming that DNA imposes a magnesium limitation stress (Table 2). Biofilms that were exposed to DNA during either the cultivation or challenge stages only, showed similar resistance profiles to biofilms grown and challenged in magnesium-replete conditions (Tables 1–2). Therefore, the DNA-induced resistance of biofilms requires both the cultivation and challenge under cation-limiting conditions. These latter two observations rule out the possibility that negatively charged DNA simply interacts with cationic antimicrobial peptides and prevents their access to bacterial cells.

**Table 2:** DNA-induced resistance of biofilms requires both cultivation and challenge under cation-limiting conditions.

Strain	Cultivation conditions^a^	Challenge conditions^a^	MBEC^b^	
			PxnB	Coln
PAO1	2 mM Mg^2+^	2 mM Mg^2+^, 0.75% DNA	640	640
PAO1	20 µM Mg^2+^	20 µM Mg^2+^	2560	2560
PAO1	2 mM Mg^2+^, 0.75% DNA	2 mM Mg^2+^	640	320

a Biofilms were cultivated and challenged in BM2 defined medium containing different Mg^2+^ concentrations with or without DNA, as indicated.

b The minimum biofilm eradication concentration (MBEC) is defined as the lowest concentration of antibiotic required for complete killing of the surface-adhered biofilm. The MBEC values shown are the median values from four replicate experiments.

The minimum inhibitory concentration (MIC) is defined as the lowest concentration of antibiotic required to inhibit the growth of planktonic cells that were shed from the peg.

PxnB  =  polymyxin B; Coln  =  colistin.

## Discussion

Infections caused by *P. aeruginosa* continue to be a leading cause of mortality among immunocompromised patients. The ability of *P. aeruginosa* to form biofilms promotes survival of the bacteria in the presence of antimicrobials and host defense mechanisms and is thought to contribute significantly to its ability to survive long-term within the hostile environment of chronically-infected patients. Understanding the mechanisms underlying antibiotic resistance and especially biofilm-specific antimicrobial resistance is of significant importance in the development of new treatment options and/or strategies.

We have identified a novel mechanism of biofilm-associated antibiotic resistance in which the presence of DNA in the extracellular matrix of biofilms creates a localized cation-limited environment that is detected by *P. aeruginosa* leading to the induction of LPS modification genes and resistance to antimicrobials. Magnesium limitation has long been known as an *in vitro* signal that induces resistance to CAPs in *P. aeruginosa* [59]. As an intracellular pathogen, the PhoPQ system of *Salmonella typhimurium* is activated by limiting magnesium *in vitro* and *phoP*-regulated genes are also induced after invasion of macrophages and epithelial cells [65]. These observations suggested that Mg^2+^ is limiting within host cells, but it was recently shown that vacuole acidification and low pH is the crucial environmental trigger of PhoPQ activation [66]. Many extracellular pathogens possess homologs of the cation-sensing PhoPQ TCS that responds to magnesium limitation and induces genes necessary for surviving this environmental challenge [65]. However, to date the identification of a relevant *in vivo* environment for *P. aeruginosa* which is cation limited has remained elusive. We have demonstrated that DNA-rich environments, such as biofilms, are cation limited.

While Mg^2+^ limitation has been identified as a signal involved in induced resistance to aminoglycosides in *P. aeruginosa* [59], the contribution of the PhoPQ-regulated LPS modifications has not been clearly determined. *PhoQ* mutants, which constitutively express *phoP* and are constitutively resistant to cationic antimicrobial peptides, are also more resistant to aminoglycosides [43]. In *S. typhimurium*, PhoPQ regulates multiple LPS modifications that decrease the OM permeability to membrane cationic dyes, bile salts and antibiotics, including gentamicin [67]. We report here that DNA-induces aminoglycoside resistance in *P. aeruginosa* biofilms, and this resistance is partially dependent on the LPS modification operon *PA3552–PA3559*. The aminoarabinose modification likely blocks the self-promoted uptake of aminoglycosides, which normally bind and displace cations that crosslink adjacent LPS molecules [68].

Previous reports have documented the involvement of *P. aeruginosa* PmrAB [49] and the *E. coli* PmrAB homologs BasRS [69] in regulating the formation of an antimicrobial peptide-tolerant subpopulation within biofilms. In pure culture *P. aeruginosa* biofilms, genomic DNA localizes throughout the biofilm surface monolayer and surrounds the mushroom-shaped microcolonies [51]. This coincides with the localization of a CAP-tolerant subpopulation of bacteria that expresses the *PA3552–PA3559* operon along the surface of mushroom-structured *P. aeruginosa* biofilms [49]. To date, it was thought unlikely that a biofilm environment may be cation limited. However, our data indicates that the presence of DNA in biofilms does indeed result in a cation-limited environment, resulting in the induction of the LPS modification operon *PA3552–PA3559*.

To our knowledge this is the first report to identify the antimicrobial properties of DNA. Above certain concentrations (∼0.5% (w/v)) extracellular DNA inhibited planktonic growth and biofilm formation. Recently, a novel host defense mechanism was discovered whereby stimulated neutrophils ejected a mesh-like net of intracellular DNA and proteins that functions to trap and kill pathogens [70]. The antimicrobial property of neutrophil nets was attributed to DNA-associated histones and other antimicrobial peptides [70]. However, our results demonstrate that above certain concentrations, the DNA itself is antimicrobial due to cation chelation. In principle, cation chelation by DNA is similar to another recently identified host defense mechanism, where the Mn^2+^ and Zn^2+^ metal chelation properties of the host innate-immune protein calprotectin was shown to limit *Staphylococcus aureus* growth in tissue abscesses [71].

Staining of peg-adhered biofilms indicated that DNA was present throughout the biofilm. (Fig 5B). This data supports the hypothesis that the release of genomic DNA by lysed cells following exposure to inhibitory concentrations of extracellular DNA may result in a continual release of DNA by dying cells and a DNA gradient within the biofilm. Our observation that DNA imposes a cation gradient in biofilm is also consistent with previous reports of oxygen and nutrient gradients within biofilms, which result in diverse physiological cellular states within a biofilm community [72].

Although DNA is toxic at high concentrations, it functions as a double-edged sword whereby sub-inhibitory DNA concentrations serve to protect bacteria from antibiotic exposure, either from the host immune response or from antimicrobial treatment. It has previously been reported that Mg^2+^ concentrations within the airway surface fluid are high (2.2 mM) [73],[74]. However, sputum samples from the lungs of CF patients have very high concentrations of DNA, up to 20 mg/ml (2% (w/v)) [75],[76]. It is likely that within the CF lung, localized cation limited environments exist within DNA-rich microcolonies. It is also known that CF airway fluid contains high levels of neutrophil defensins [77] and that sub-lethal doses of CAPs induce *PA3553* gene expression, although independently of PhoPQ and PmrAB [45]. Therefore, it appears that there are multiple environmental signals in the CF lung that can induce the expression the *PA3552–PA3559* operon, which may explain why many *P. aeruginosa* CF isolates show LPS modifications such as aminoarabinose addition to lipid A [78]. As many *P. aeruginosa* strains isolated from the CF lung overproduce the negatively charged EPS alginate, we hypothesized that alginate may also be a relevant *in vivo* signal inducing expression of the *PA3552–PA3559* operon. However, induction of *PA3553* gene expression does not occur in the presence of alginate (data not shown).

The observation that DNA is present in the lungs of CF patients has prompted the use of DNAseI as a therapeutic agent to reduce the sputum viscosity and improve lung function [75],[76]. However, our data suggests that the success of DNAseI therapy may, in part, be attributed to the degradation of DNA and subsequent disarming of the PhoPQ/PmrAB response and antibiotic resistance mechanisms. While previous studies have shown the biofilm matrix to function as a diffusion barrier to antibiotics, these results demonstrate a novel function of the biofilm matrix component DNA, where the cation chelating properties of DNA in biofilms induces resistance to host-derived or therapeutic antimicrobials. Furthermore, these findings indicate that DNA-rich environments, such as bacterial biofilms or the CF lung, may represent the natural setting where bacterial growth is cation limited, and highlight the importance of the PhoPQ/PmrAB controlled response and LPS modifications in antibiotic resistance in biofilms.

## Materials and Methods

### Bacterial strains

*Pseudomonas aeruginosa* PAO1 and *lux*-tagged PAO1::p16S*lux* [79] were used as wild-type strains. The mini-Tn5-*lux* transposon mutant in the CAP resistance gene *PA3553::lux (arnC)* was previously constructed [47]. For all experiments involving DNA, DNA was isolated in the absence of EDTA and resuspended in the buffer or media in which each experiment was carried out.

### Growth inhibition, growth restoration and killing assays

Growth kinetics of *P. aeruginosa* was carried out in LB or BM2 media [47] supplemented with low molecular weight salmon sperm (Fluka) or *P. aeruginosa* genomic DNA, with and without the addition of various cations in excess (25, 12.5, 6.25, and 3.125 mM). Cation sources were MgCl_2_, CaCl_2_, MnCl_2_ and ZnCl_2_. For Mg^2+^ supplementation, no difference was observed when MgCl_2_ was substituted with MgSO_4_. Growth assays were carried out in 100 µl volumes in transparent 96-well plates (Nunc). Fifty µl of sterile mineral oil was added to each well to prevent evaporation during the assay. Microplate planktonic cultures were incubated at 37°C in a Wallac Victor^3^ luminescence plate reader (Perkin-Elmer) and optical density (growth, OD600) readings were taken every 20 minutes throughout growth. Killing assays were carried out as previously described [80]. Briefly, overnight cultures of PAO1::p16S*lux* were washed and diluted in 25 mM sodium phosphate or 50 mM Hepes buffer, pH 7.4, as indicated in the figure legends. 5×10^7^ cfu were exposed to varying concentrations of salmon sperm DNA, in the presence or absence of excess cations, and CPS monitored over time, as a measure of viability. Each growth or killing experiment was performed at least five times and representative curves are shown.

### Cultivation and imaging of peg adhered biofilms

For microscopy analysis of peg-adhered biofilms, PAO1 was cultivated on pegs (NUNC-TSP), washed as described below and stained with 1 µM 7-hydroxy-9H-(1,3-dichloro-9,9-dimethylacridin-2-one) (DDAO) (Molecular Probes) or 10 µM propidium iodide (PI) for 10 mins. Individual pegs were removed and placed on a drop of 0.9% saline on a glass slide prior to visualization. Images were captured with a Leica DMIREB2 inverted, epifluorescence microscope.

### DNA lysis, live cell imaging and *P. aeruginosa* specific semi-quantitative PCR

For DNA lysis experiments, overnight cultures of PAO1 producing mCherry fluorescent lipoproteins with sorting signals for either the OM (lipoCSFP-ChFP) or IM (lipoCKVE-ChFP) were subcultured 1/100 and grown for 3 h to mid-log phase (OD 0.5) [60],[61]. Overnight cultures were diluted 1 in 100 and grown to mid-log phase. 1.5×10^8^ cells were spun, washed in sodium phosphate buffer (25 mM, pH 7.4) and resuspended in 50 µl of 1 mM EDTA, 2% (w/v) salmon sperm DNA or buffer alone (negative control). Cells were lysed for 10 mins, pelleted (8000 rpm, 5 mins) and 1 µl of supernatent used as a template for semi-quantitative PCR (25 cycles). PCR was carried out on lysates obtained from 2% (w/v) DNA, 1 mM EDTA and untreated control cells. For *P. aeruginosa* specific PCR studies, 1 µl of the lysate was used as a template for semi-quantitative PCR using *P. aeruginosa* specific primers (F-5′ gaggatcccgccgggttttttgtgtctg-3′, R-5′gaggatcccaggagtgatattagcgattc-3′). These primers amplify a 216 bp product corresponding to the promoter region of the *rsmZ* gene in *P. aeruginosa*. PCR controls included 2% (w/v) salmon sperm DNA alone to ensure the specificity of the primers for *P. aeruginosa* DNA, a negative PCR control with no template and a positive PCR control containing *P. aeruginosa* genomic DNA as template.

For microscopy, cells were washed, concentrated 2.5 fold in sodium phosphate buffer (25 mM, pH 7.4) and stained with 10 µM PI or left unstained. Cells were visualized on agarose beds with a Leica DMIREB2 inverted microscope equipped with an ORCA-ER digital camera and Openlab software (Improvision). For DNAse treatment of DNA/EDTA lysed cells or relevant controls, cells were treated with DNAseI (500 µg/ml) for 45 mins at 37°C prior to the addition of fluorescent dyes and microscopic analysis.

### Measurement of chelating ability of DNA

To determine the specificity of cation chelation by DNA, 1.25% (w/v) salmon sperm DNA was resuspended in 50 mM Hepes buffer, pH 7.4, and incubated individually with 2.5 mM Mg^2+^, Ca^2+^, Zn^2+^ or Mn^2+^ or a cocktail of 0.625 mM of each cation. After 3 h incubation at room temperature with constant rotation, samples were centrifuged in a Amicon ultra column (Millipore) with a 10 kDa cutoff (3200 g for 30 mins). All unbound cations passed through the filter but DNA was retained. The filtrate was sent for flame atomic absorption spectroscopy analysis to determine the percentage of cation not bound by DNA (Bodycote Testing Group, Portland, OR, USA). Values represented are the percent of cations bound to DNA.

### Real-time gene expression in planktonic and biofilm cultures

Overnight cultures were grown in LB medium or BM2 defined medium (20 mM succinate) supplemented with 2, 1 or 0.02 mM Mg^2+^ and extracellular DNA as indicated, diluted 1/100 into 100 µl of culture medium in 96-well black plates with a transparent bottom (9520 Costar; Corning Inc.) and overlayed with 50 µl of mineral oil to prevent evaporation. Microplate planktonic cultures were incubated at 37°C in a Wallac Victor^3^ luminescence plate reader (Perkin-Elmer) and optical density (growth, OD600) and luminescence (gene expression, CPS) readings were taken every 20 minutes throughout growth. For DNAse treatment experiments, 2% salmon sperm DNA was treated for 48 hrs at 37°C with 500 µg/ml of DNAseI enzyme in 40 mM Tris, 10 mM MgSO_4_ and CaCl_2_. Biofilms were cultivated on 96-well format, polystyrene pegs (Nunc-TSP) that were immersed in 200 µl of growth medium. After biofilm cultivation, non-adherent cells were removed by rinsing the pegs in 0.9% NaCl. Gene expression in peg-adhered biofilms was measured by luminescence readings in the Wallac MicroBeta Trilux multi-detector (Perkin-Elmer). Biofilm formation on the pegs was quantitated by crystal violet (CV) staining (OD600) as previously described [81].

### Antimicrobial susceptibility testing in biofilms

*P. aeruginosa* biofilms were tested for susceptibility using the Calgary Biofilm Device protocol [6]. Overnight cultures of *P. aeruginosa* PAO1 and *PA3553::lux* were grown in BM2 defined medium with magnesium concentrations as indicated and supplemented with 0.75% (w/v) salmon sperm DNA. This concentration of DNA was not toxic in BM2 medium with 2 mM Mg^2+^. Starter cultures were diluted in the appropriate medium and inoculated at a concentration of 1.5×10^6^ cfu/well. Biofilms were cultivated on the peg lids by shaking the plate at 37°C for 24 hours. The pegs were rinsed twice in 0.9% NaCl and transferred to challenge plates, which consisted of a serial two-fold dilution gradient of polymyxin B, colistin, gentamycin or tobramycin. Peg-adhered biofilms were challenged in the same media in which they were cultivated. Following a 24-hour antibiotic challenge, the MIC values were determined by measuring growth (OD600) in the challenge plate. After biofilm challenge, the surviving cells in peg-adhered biofilms were rinsed twice in 0.9% NaCl, DNAseI treated (25 µg/ml) for 30 mins and sonicated for 10 mins to remove attached cells. The surviving cells were enumerated by serial dilution and plate counts to determine the MBEC value.

## Supporting Information

Figure S1 DNA released from lysed cells forms a mesh-like coating surrounding and connecting individual cells. Propidium iodide (PI) staining of DNA or EDTA lysed cells and relevant controls in the absence and presence of DNAse treatment.
(10.27 MB TIF)
