# Supplementary figures and images for: Extracellular DNA Chelates Cations and Induces Antibiotic Resistance in Pseudomonas aeruginosa Biofilms
Source: PLoS Pathog. 2008 Nov 21;4(11):e1000213. doi: 10.1371/journal.ppat.1000213 (PMC2581603; doi:10.1371/journal.ppat.1000213)

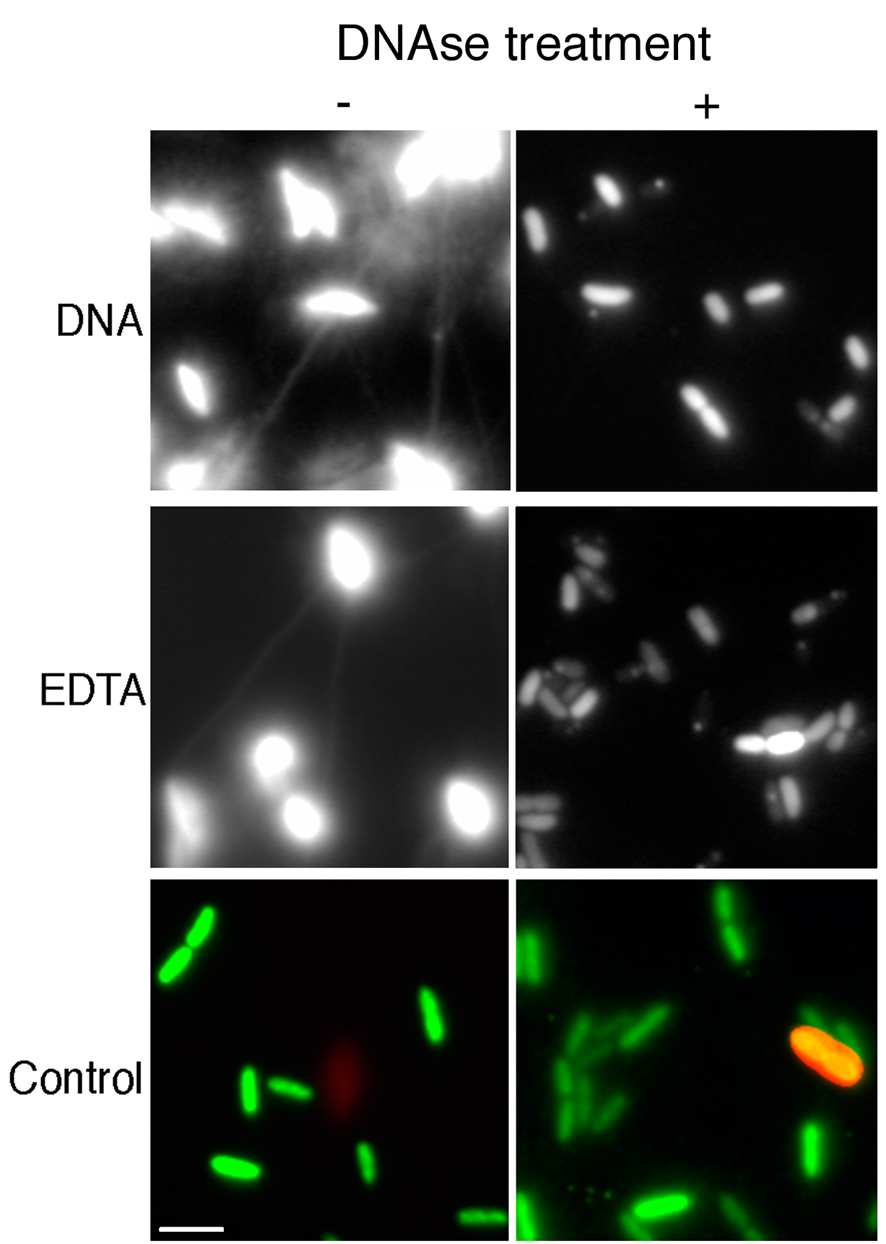

Supplement: Figure S1 — DNA released from lysed cells forms a mesh-like coating surrounding and connecting individual cells. Propidium iodide (PI) staining of DNA or EDTA lysed cells and relevant controls in the absence and presence of DNAse treatment. (10.27 MB TIF) [file ppat.1000213.s001.tif]
